# Minor Components of Micropapillary and Solid Subtypes in Lung Adenocarcinoma are Predictors of Lymph Node Metastasis and Poor Prognosis

**DOI:** 10.1245/s10434-015-5043-9

**Published:** 2016-02-02

**Authors:** Yue Zhao, Rui Wang, Xuxia Shen, Yunjian Pan, Chao Cheng, Yuan Li, Lei Shen, Yang Zhang, Hang Li, Difan Zheng, Ting Ye, Shanbo Zheng, Yihua Sun, Haiquan Chen

**Affiliations:** Department of Thoracic Surgery, Fudan University Shanghai Cancer Center, Shanghai, China; Department of Oncology, Shanghai Medical College, Fudan University, Shanghai, China; Department of Pathology, Fudan University Shanghai Cancer Center, Shanghai, China; Shanghai Chest Hospital, Shanghai Jiao Tong University, Shanghai, China; Institutes of Biomedical Sciences, Fudan University, Shanghai, China

## Abstract

**Background:**

Lung adenocarcinoma with micropapillary and solid predominant subtypes was reported to be associated with poor prognosis; however, whether minor components (non-predominant) of micropapillary and solid subtypes predict poor prognosis remains unknown. In this study, we investigated the predictive and prognostic value of lymph node metastasis of minor micropapillary and solid components.

**Methods:**

Specimens of resected tumors of 1244 patients were reclassified to determine the predominant subtype and minor components (>5 %, but not predominant). Of these specimens, 105 contained a micropapillary component and 210 contained a solid component. The correlation between each subtype and lymph node metastasis was analyzed, and survival analyses were used to determine the association between each subtype and patient survival.

**Results:**

Adenocarcinomas harboring micropapillary and/or solid components held higher rates of metastatic lymph node stations (25.2 % vs. 15.6 %, *p* = 0.002; and 24.0 % vs. 14.9 %, *p* < 0.001, respectively) and lymph nodes (17.3 % vs. 10.1 %, *p* = 0.004; and 15.5 % vs. 9.7 %, *p* = 0.001, respectively). Patients with micropapillary and solid components in their tumors showed a shorter median recurrence-free survival (15.8 vs. 62.8 months, *p* < 0.001; and 20.8 months vs. not reached, *p* < 0.001) and overall survival (47.0 months vs. not reached, *p* < 0.001; and 69.0 months vs. not reached, *p* < 0.001).

**Conclusions:**

Minor components of micropapillary and/or solid subtypes of lung adenocarcinoma are correlated with lymph node metastasis and poor prognosis. Thus, it is beneficial to focus not only on predominant subtypes but also minor components to predict prognoses and make therapeutic strategies more comprehensively.

**Electronic supplementary material:**

The online version of this article (doi:10.1245/s10434-015-5043-9) contains supplementary material, which is available to authorized users.

Adenocarcinoma has long been an independent histological class of lung cancer and has been broadly studied for therapeutic efficacy and toxicities.^1^–^5^ In 2011, a new classification system of subtypes of lung adenocarcinoma was recommended by the International Association for the Study of Lung Cancer (IASLC), American Thoracic Society (ATS), and European Respiratory Society (ERS) to further study and advance the field.^6^ Since then, a number of investigations have provided evidence for the relationship between different subtypes and treatment outcomes.^7^–^11^

Several studies have reported that micropapillary- and solid-predominant subtypes of lung adenocarcinoma were associated with poor prognoses;^12^–^16^ however, lung adenocarcinomas usually contain complex mixtures of different subtypes.^17^ Whether minor components of each subtype are associated with lymph node metastasis and poor prognosis still remains unknown and needs further clarification.

In this study, we comprehensively analyzed 1244 consecutive patients who were diagnosed with stage I–IV invasive lung adenocarcinoma and who underwent surgical resection between August 2006 and May 2013. Our aim was to provide clinical evidence for the predictive and prognostic value of minor components of lung adenocarcinoma.

## Patients and Methods

### Patients and Tissues

Overall, 1244 consecutive patients who were diagnosed with invasive lung adenocarcinoma and who underwent surgical resection between August 2006 and May 2013 were included in this study. R0 resection was achieved in 1240 of the 1244 patients. Patients with no or insufficient archived tumor specimens were excluded, and no patient underwent neoadjuvant therapy. To ensure an accurate assessment, tumors were reclassified by three pathologists (XS, LS, and YL) and categorized into the following subtypes: lepidic (L), acinar (A), papillary (P), micropapillary (M), and solid (S) predominant subtypes, as well as invasive mucinous adenocarcinoma (IMA), according to the newly announced IASLC/ATS/ERS lung adenocarcinoma classification system.^6^ Each of the 1244 slides was reviewed by these three pathologists separately. Discordant results were reconsidered together by the three pathologists until a consensus was reached. Specimens that did not belong to any one of these categories were marked as ‘others’. For those specimens that were mixed by more than one subtype, the subtype that occupied most of the tumor (even if <50 %) was defined as the predominant subtype, and subtype(s) that occupied no less than 5 % but were not predominant were defined as minor components. We put them in a sequence from the largest to the smallest amount.

This study was approved by the Committee for Ethical Review of Research (Fudan University Shanghai Cancer Center IRB# 090977-1).

### Statistical Analysis

Clinical and pathological characteristics, such as sex, age, smoking status, lymphovascular invasion status, tumor location, and nodal status (N0, N1, or N2), together with predominant subtypes and minor components, were analyzed to determine the correlation with lymph node metastasis. *p* values, hazard ratios and 95 % confidence intervals (CI) were calculated using Pearson’s Chi square test or Fisher’s exact test, and the two-tailed significance level was set at 0.05. Metastatic rates of lymph node stations and lymph nodes of each predominant subtype and minor component were calculated to evaluate the correlation between lymph node metastasis and each subtype. *p* values were calculated using Student’s *t* test. Moreover, Kaplan–Meier survival curves were used to compare patients’ recurrence-free survival (RFS) and overall survival (OS). Finally, a multivariate Cox model adjusted for sex, age, smoking status, lymphovascular invasion status, tumor location, and nodal status (N0, N1, or N2) was used to determine the odds ratio (OR) and 95 % CI for each minor component. All statistical analyses were performed using SPSS version 20.0 (IBM Corporation, Armonk, NY, USA).

## Results

For the 1244 patients with pathologically validated lung adenocarcinoma included in this study, a reclassification by three pathologists manifested that there were 158 lepidic-predominant, 598 acinar-predominant, 170 papillary-predominant, 68 micropapillary-predominant, 171 solid-predominant, and 72 invasive mucinous adenocarcinoma. Clinical and pathological characteristics are shown in Table 1. Of the 1244 patients, 109 had a tumor containing a minor component of lepidic subtype, 196 a minor acinar component, 178 a minor papillary component, 105 a minor micropapillary component, 210 a minor solid component, and 62 had a tumor containing a minor invasive mucinous component. Patients were aged 60 years (range 22–110), 560 were males and 684 were females (Table 1). The pathologic stage was 0 in 6 (0.5 %) patients, IA in 517 (41.6 %) patients, IB in 178 (14.3 %) patients, IIA in 120 (9.6 %) patients, IIB in 46 (3.7 %) patients, IIIA in 338 (27.2 %) patients, IIIB in 15 (1.2 %) patients, and IV in 24 (1.9 %) patients.

**Table 1:** Clinicopathological characteristics of different subtypes of lung adenocarcinoma (*n* = 1244)

	Total	N0 (*n* = 789)	N1/N2 (*n* = 455)	*p* value	HR (95 % CI)
Gender					
Male	560 (45.0 %)	327 (41.4 %)	233 (51.2 %)	0.001	1.282 (1.108–1.483)
Female	684 (55.0 %)	462 (58.6 %)	222 (48.8 %)		
Age					
< Average	598 (48.1 %)	360 (45.6 %)	238 (52.3 %)	0.023	1.185 (1.023–1.372)
≥ Average	646 (51.9 %)	429 (54.4 %)	217 (47.7 %)		
Smoking status					
Former/current	403 (32.4 %)	225 (28.5 %)	178 (39.1 %)	<0.001	1.340 (1.159–1.553)
Never	841 (67.6 %)	564 (71.5 %)	277 (60.9 %)		
Tumor location^a^					
LUL	330 (26.5 %)	193 (24.5 %)	137 (30.1 %)	0.033	0.838 (0.717–0.979)
LLL	200 (16.1 %)	119 (15.1 %)	81 (17.8 %)	0.208	0.885 (0.734–1.066)
RUL	406 (32.6 %)	274 (34.7 %)	132 (29.0 %)	0.038	1.186 (1.006–1.397)
RML	103 (8.3 %)	64 (8.1 %)	39 (8.6 %)	0.777	0.963 (0.743–1.248)
RLL	231 (18.6 %)	150 (19.0 %)	81 (17.8 %)	0.597	1.053 (0.868–1.277)
LVI					
Yes	203 (16.3 %)	35 (4.4 %)	168 (36.9 %)	<0.001	3.003 (2.674–3.378)
No	1041 (83.7 %)	754 (95.6 %)	287 (63.1 %)		
Differentiation					
Well	169 (13.6 %)	153 (19.4 %)	16 (3.5 %)	<0.001	0.232 (0.145–0.372)
Moderate	714 (57.4 %)	492 (62.4 %)	222 (48.8 %)	<0.001	0.707 (0.612–0.818)
Poor	361 (29.0 %)	144 (18.3 %)	217 (47.7 %)	<0.001	2.232 (1.946–2.558)
Tumor size (cm)					
< 3	756 (60.8 %)	572 (72.5 %)	184 (40.4 %)	<0.001	2.283 (1.965–2.646)
≥ 3	488 (39.2 %)	217 (27.5 %)	271 (59.6 %)		
Predominant subtype					
L	158 (12.7 %)	154 (19.5 %)	4 (0.9 %)	<0.001	0.061 (0.023–0.161)
A	598 (48.1 %)	211 (46.4 %)	387 (49.0 %)	0.363	0.935 (0.806–1.082)
P	170 (13.7 %)	98 (12.4 %)	72 (15.8 %)	0.092	1.188 (0.979–1.441)
M	68 (5.5 %)	35 (4.4 %)	33 (7.3 %)	0.035	1.353 (1.046–1.748)
S	171 (13.7 %)	62 (7.9 %)	109 (24.0 %)	<0.001	1.976 (1.715–2.278)
IMA	72 (5.8 %)	48 (6.1 %)	24 (5.3 %)	0.556	0.907 (0.648–1.267)
Minor components					
L	109 (8.8 %)	94 (11.9 %)	15 (3.3 %)	<0.001	0.355 (0.221–0.571)
A	196 (15.8 %)	123 (15.6 %)	73 (16.0 %)	0.832	1.021 (0.838–1.247)
P	178 (14.3 %)	97 (12.3 %)	81 (17.8 %)	0.008	1.297 (1.083–1.553)
M	105 (8.4 %)	49 (6.2 %)	56 (12.3 %)	<0.001	1.522 (1.252–1.852)
S	210 (16.9 %)	103 (13.1 %)	107 (23.5 %)	<0.001	1.513 (1.292–1.773)
IMA	62 (5.0 %)	34 (4.3 %)	28 (6.2 %)	0.150	1.250 (0.941–1.661)

*p* value, HR, and 95 % CI were calculated using the Chi square test and Fisher’s exact test

*HR* hazard ratio, *CI* confidence interval, *LUL* left upper lobe, *LLL* left lower lobe, *RUL* right upper lobe, *RML* right middle lobe, *RLL* right lower lobe, *LVI* lymphovascular invasion, *L* lepidic, *A* acinar, *P* papillary, *M* micropapillary, *S* solid, *IMA* invasive mucinous adenocarcinoma

^a^When adding the percentage of this category together, a number more than 100 % may be obtained as some tumors occurred in more than one lobe

### The Predictive Value of Lymph Node Metastasis

Of the 1244 patients, 789 (63.4 %) had pathologically validated N0 disease and 455 (36.6 %) had either pathologically validated N1 or N2 disease (Table 1). Our data showed that lymph node metastasis had a correlation with male sex (*p* < 0.001), younger age at initial diagnosis (*p* = 0.023), ever-smoker (*p* < 0.001), lymphovascular invasion (*p* < 0.001), poor differentiation (*p* < 0.001), and larger tumor size (*p* < 0.001). The micropapillary-predominant subtype (*p* = 0.035; HR 1.353; 95 % CI 1.046–1.748) and solid-predominant subtype (*p* < 0.001; HR 1.976; 95 % CI 1.715–2.278) were both associated with lymph node metastasis, along with the papillary minor component (*p* = 0.008; HR 1.297; 95 % CI 1.083–1.553), micropapillary minor component (*p* < 0.001; HR 1.522; 95 % CI 1.252–1.852), and solid minor component (*p* < 0.001; HR 1.513; 95 % CI 1.292–1.773). On the other hand, tumors presenting as well-differentiated (*p* < 0.001) or moderately differentiated (*p* < 0.001), lepidic-predominant subtype (*p* < 0.001), and lepidic minor component (*p* < 0.001) showed lower probabilities of having lymph node metastasis (Table 1).

To further investigate the correlation between different subtypes and lymph node metastasis, we also recorded the number of metastatic lymph node stations/lymph nodes and resected lymph node stations/lymph nodes, and made a calculation of metastatic rates (Tables 2, 3). Regional lymph node stations were defined as per the recommendations made by Mountain and Dresler in 1997.^18^ According to our data, tumors harboring the micropapillary minor component and solid minor component had higher metastatic rates of lymph node station (25.2 vs. 15.6 %, *p* = 0.002; and 24.0 vs. 14.9 %, *p* < 0.001, respectively) and lymph node (17.3 vs. 10.1 %, *p* = 0.004; and 15.5 vs. 9.7 %, *p* = 0.001, respectively). A similar trend was observed in tumors with micropapillary-predominant and solid-predominant subtypes (Tables 2, 3). Additionally, we found that patients with lepidic-predominant or minor component tumors had a lower probability of experiencing lymph node metastasis (also shown in Tables 2, 3). Furthermore, we also analyzed the relationship between the second predominant subtype and lymph node metastasis. All 606 patients with two or more presenting subtypes were included (see electronic supplementary Table 1), and this analysis showed a similar result as previous ones (see electronic supplementary Tables 2 and 3).

**Table 2:** Relationship between different subtypes of lung adenocarcinoma and metastatic rate of lymph node station (*n* = 1244)

Subtype	Negative (%)^a^	Minor component (%)	*p* value	Predominant (%)	*p* value
L	17.5	5.2	<0.001	8.2	<0.001
A	16.6	15.6	0.650	15.5	0.233
P	15.9	19.5	0.106	20.1	0.081
M	15.6	25.2	0.002	23.8	0.066
S	14.9	24.0	<0.001	28.7	<0.001
IMA	16.2	21.4	0.138	14.7	0.580

Metastatic rate of lymph node station = (number of metastatic lymph node stations/number of totally resected lymph node stations) × 100 %

*L* lepidic, *A* acinar, *P* papillary, *M* micropapillary, *S* solid, *IMA* invasive mucinous adenocarcinoma

^a^Percentage of patients with subtype of interest not observed or less than 5 %

**Table 3:** Relationship between different subtypes of lung adenocarcinoma and metastatic rate of lymph node (*n* = 1244)

Subtype	Negative (%)^a^	Minor component (%)	*p* value	Predominant (%)	*p* value
L	11.4	3.1	<0.001	6.3	<0.001
A	10.8	10.1	0.663	9.8	0.176
P	10.5	11.9	0.397	13.5	0.081
M	10.1	17.3	0.004	17.5	0.056
S	9.7	15.5	0.001	18.0	<0.001
IMA	10.6	12.7	0.441	9.7	0.687

Metastatic rate of lymph node = (number of metastatic lymph nodes/number of totally resected lymph nodes) × 100 %

*L* lepidic, *A* acinar, *P* papillary, *M* micropapillary, *S* solid, *IMA* invasive mucinous adenocarcinoma

^a^Percentage of patients with subtype of interest not observed or less than 5 %

### Does Each Subtype Predict a Different Prognosis?

**Fig. 1:**
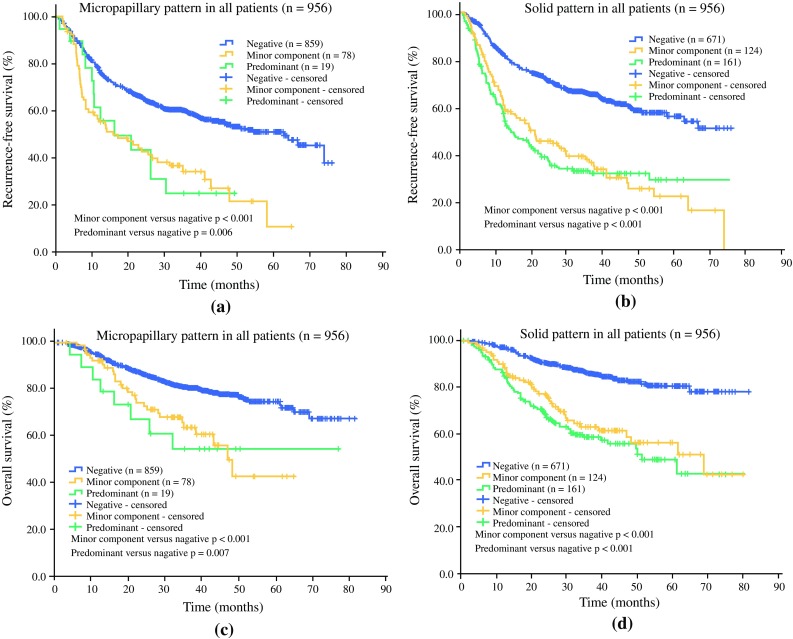
Kaplan–Meier survival curves of RFS and OS for patients with micropapillary and solid subtypes. **a** RFS for patients with micropapillary-predominant tumors and tumors containing minor micropapillary components; **b** RFS for patients with solid-predominant tumors and tumors containing minor solid components; **c** OS for patients with micropapillary-predominant tumors and tumors containing minor micropapillary components; **d** OS for patients with solid-predominant tumors and tumors containing minor solid components. A two-tailed *p* < 0.05 was regarded as statistically different. Negative refers to the percentage of patients with subtype of interest not observed or < 5 %. *RFS* recurrence-free survival, *OS* overall survival

Of the 1244 patients, 288 lacked sufficient follow-up data; therefore, we included the remaining 956 patients in subsequent survival analysis. Seventy-eight of the 956 patients had a tumor containing a minor micropapillary component, and 124 had a tumor containing a minor solid component, along with 19 micropapillary-predominant subtypes and 161 solid-predominant subtypes (Fig. 1).

In univariate analysis, patients with tumors containing a minor component of micropapillary subtype had a significantly shorter RFS (*p* < 0.001) and OS (*p* < 0.001). A similar observation was made among patients with tumors containing a minor component of solid subtype, which also showed a significantly shorter RFS (*p* < 0.001) and OS (*p* < 0.001). Meanwhile, patients with tumors of micropapillary- or solid-predominant subtype also had significantly worse RFS (*p* = 0.006 and *p* < 0.001, respectively) and OS (*p* = 0.007 and *p* < 0.001, respectively).

In a multivariate analysis using the Cox model adjusted for sex, age, smoking status, lymphovascular invasion status, tumor location, and nodal status (N0, N1, or N2), we found that tumors with a minor component of micropapillary subtype were correlated with shorter RFS (*p* < 0.001; OR 0.524; 95 % CI 0.388–0.708) and OS (*p* = 0.012; OR 0.585; 95 % CI 0.385–0.890) (Table 4). Tumors with a minor component of solid subtype were also related to a worse RFS (*p* = 0.014; OR 0.728; 95 % CI 0.567–0.936) and OS (*p* = 0.039; OR 0.690; 95 % CI 0.484–0.982) (Table 4). No significant difference was observed between other minor components and patient survival.

**Table 4:** Multivariate analysis of recurrence-free survival and overall survival for patients with minor components of different subtypes of lung adenocarcinoma

Subtype	OR	95 % CI	*p* value
Recurrence-free survival			
Lepidic	1.361	0.905–2.041	0.139
Acinar	1.104	0.83–1.453	0.485
Papillary	0.978	0.752–1.271	0.866
Micropapillary	0.524	0.388–0.708	<0.001
Solid	0.728	0.567–0.936	0.014
Invasive mucinous carcinoma	1.351	0.883–2.066	0.165
Overall survival			
Lepidic	1.730	0.878–3.413	0.113
Acinar	1.122	0.754–1.669	0.571
Papillary	1.284	0.861–1.912	0.220
Micropapillary	0.585	0.385–0.890	0.012
Solid	0.690	0.484–0.982	0.039
Invasive mucinous carcinoma	0.870	0.519–1.456	0.594

Multivariate Cox model was adjusted for sex, age, smoking status, lymphovascular invasion or no lymphovascular invasion, tumor location, and nodal status (N0, N1, or N2). A two-tailed *p* < 0.05 was regarded as statistically different

*OR* odds ratio, *CI* confidence interval

## Discussion

Lymph node metastasis is a major way for cancer cells to migrate from the primary tumor to distant organs, which promises a poor prognosis for lung cancer patients. With the classification of subtyping lung adenocarcinoma by the IASLC/ATS/ERS,^6^ several studies have reported that micropapillary- and solid-predominant subtypes were related to poor prognoses;^9^,^12^–^14^,^19^–^21^ however, lung adenocarcinomas usually contain a mixture of different subtypes. Thus, it is necessary for clinicians and researchers to understand whether minor components influence patients’ prognoses to help predict their prognoses and make therapeutic strategies. Yeh et al. pointed out that the presence of micropapillary or solid patterns were of predictive value with increased risk of occult lymph node metastasis;^22^ however, studies focusing on minor components are limited. In this study, our aim was to determine whether there was a correlation between minor components of lung adenocarcinoma and lymph node metastasis. According to our study of 1244 patients with pathologically proven lung adenocarcinoma that was initially diagnosed between August 2006 and May 2013, we found that patients with lung adenocarcinoma of lepidic (*p* < 0.001) subtype tended to have a lower rate of lymph node metastasis, while patients with lung adenocarcinoma of papillary, micropapillary and solid subtypes were more likely to have lymph node metastasis (Table 1). These results are consistent with several previous studies^19^–^21^,^23^,^24^ but none reported the results of patients with lung adenocarcinoma of the papillary subtype. In addition, our data showed that patients with lung adenocarcinoma predominated by different subtypes, as well as patients presenting with different minor components, experience different possibilities of presenting with lymph node metastasis.

To further understand the relationship between different subtypes and lymph node metastasis, we recorded the number of metastatic lymph node stations/lymph nodes and resected lymph node stations/lymph nodes for each predominant subtype and minor component, and calculated the metastatic rate. According to our study, tumors with lepidic components had a lower rate of metastatic lymph node stations and lymph nodes, while tumors with micropapillary and solid components had a higher metastatic rate of lymph node stations and lymph nodes. Similar conclusions can also be made for patients with lepidic-, micropapillary- and solid-predominant and second predominant tumors (Tables 2, 3; electronic supplementary Tables 2, 3), with only one exception—the correlation between micropapillary second predominant subtype and metastatic rate of lymph node station, probably due to the sample size. That is to say, tumors containing minor components of micropapillary and solid subtypes are aggressive enough and have the potential for lymph node metastasis.

In univariate analysis, patients presenting with micropapillary and solid subtypes had shorter RFS and OS (Fig. 1). Interestingly, whether tumors contained a minor or predominant component of micropapillary and solid subtypes was not significantly different in RFS (*p* = 0.973 and *p* = 0.635, respectively, data not shown) or OS (*p* = 0.692 and *p* = 0.291, respectively, data not shown). This finding suggests that minor components of micropapillary and solid subtypes are as valuable as micropapillary- and solid-predominant subtypes in predicting patients’ prognosis. In multivariate analysis, minor components of micropapillary and solid subtypes were both independent predictive factors of a poor prognosis (Table 4). These results support the fact that micropapillary and solid subtypes are poor indicators for patients’ prognosis, even if they are minor components of a specific tumor.

## Conclusions

Minor components, as well as predominant subtypes of micropapillary and solid subtypes of lung adenocarcinoma, are independent poor indicators of lymph node metastasis and prognosis. Our study concentrates not only on the predominant subtype but also on minor components of lung adenocarcinoma. It is believed that more data are needed to better clarify this issue, and we hope that minor components of lung adenocarcinoma are taken into consideration by clinicians when predicting patients’ prognosis and designing comprehensive therapeutic strategies in order to benefit more patients.

## Electronic supplementary material

Below is the link to the electronic supplementary material.

Supplementary material 1 (DOCX 13 kb)

Supplementary material 2 (DOCX 14 kb)

Supplementary material 3 (DOCX 13 kb)
