# Supplementary material for: Minor Components of Micropapillary and Solid Subtypes in Lung Adenocarcinoma are Predictors of Lymph Node Metastasis and Poor Prognosis
Source: Ann Surg Oncol. 2016 Feb 2;23:2099–105. doi: 10.1245/s10434-015-5043-9 (PMC4858562; doi:10.1245/s10434-015-5043-9)
Supplement: Supplementary file 1 — Supplementary material 1 (DOCX 13 kb) [file 10434_2015_5043_MOESM1_ESM.docx]

Supplementary table 1. Patients with different second predominant subtypes (n = 606).

| Second predominant subtype | Number (percentage) |
| --- | --- |
| Lipidic | 83 (13.7%) |
| Acinar | 183 (30.2%) |
| Papillary | 145 (23.9%) |
| Micropapillary | 58 (9.6%) |
| Solid | 121 (20.0%) |
| Invasive mucinous adenocarcinoma | 16 (2.6%) |
