# Supplementary material for: Minor Components of Micropapillary and Solid Subtypes in Lung Adenocarcinoma are Predictors of Lymph Node Metastasis and Poor Prognosis
Source: Ann Surg Oncol. 2016 Feb 2;23:2099–105. doi: 10.1245/s10434-015-5043-9 (PMC4858562; doi:10.1245/s10434-015-5043-9)
Supplement: Supplementary file 2 — Supplementary material 2 (DOCX 14 kb) [file 10434_2015_5043_MOESM2_ESM.docx]

Supplementary table 2. Relationship between second predominant subtypes of lung adenocarcinoma and metastatic rate of lymph node station (n = 606).

Abbr.: L, lepidic; A, acinar; P, papillary; M, micropapillary; S, solid; IMA, invasive mucinous adenocarcinoma

| Subtype | Negative* | Second predominant | p |
| --- | --- | --- | --- |
| L | 19.2% | 5.7% | < 0.001 |
| A | 18.0% | 15.8% | 0.355 |
| P | 17.0% | 18.5% | 0.581 |
| M | 16.8% | 22.5% | 0.133 |
| S | 15.6% | 24.5% | 0.004 |
| IMA | 17.5% | 13.1% | 0.529 |

Metastatic rate of lymph node station = (number of metastatic lymph node stations / number of totally resected lymph node stations) * 100%

*, Percentage of patients with subtype of interest not observed or less than 5%.
