# Supplementary material for: Minor Components of Micropapillary and Solid Subtypes in Lung Adenocarcinoma are Predictors of Lymph Node Metastasis and Poor Prognosis
Source: Ann Surg Oncol. 2016 Feb 2;23:2099–105. doi: 10.1245/s10434-015-5043-9 (PMC4858562; doi:10.1245/s10434-015-5043-9)
Supplement: Supplementary file 3 — Supplementary material 3 (DOCX 13 kb) [file 10434_2015_5043_MOESM3_ESM.docx]

Supplementary table 3. Relationship between second predominant subtypes of lung adenocarcinoma and metastatic rate of lymph node (n = 606).

Abbr.: L, lepidic; A, acinar; P, papillary; M, micropapillary; S, solid; IMA, invasive mucinous adenocarcinoma

| Subtype | Negative* | Second predominant | p |
| --- | --- | --- | --- |
| L | 12.3% | 3.3% | < 0.001 |
| A | 11.5% | 10.3% | 0.524 |
| P | 11.1% | 11.1% | 0.995 |
| M | 10.4% | 17.5% | 0.041 |
| S | 10.1% | 15.1% | 0.027 |
| IMA | 11.2% | 7.1% | 0.437 |

Metastatic rate of lymph node = (number of metastatic lymph nodes / number of totally resected lymph nodes) * 100%

*, Percentage of patients with subtype of interest not observed or less than 5%.
